# Intelligent Measuring of the Volume Fraction Considering Temperature Changes and Independent Pressure Variations for a Two-Phase Homogeneous Fluid Using an 8-Electrode Sensor and an ANN

**DOI:** 10.3390/s23156959

**Published:** 2023-08-05

**Authors:** Ramy Mohammed Aiesh Qaisi, Farhad Fouladinia, Abdulilah Mohammad Mayet, John William Grimaldo Guerrero, Hassen Loukil, M. Ramkumar Raja, Mohammed Abdul Muqeet, Ehsan Eftekhari-Zadeh

**Affiliations:** 1 Department of Electrical and Electronics Engineering, College of Engineering, University of Jeddah, Jeddah 21589, Saudi Arabia; dgaisi@uj.edu.sa; 2 Faculty of engineering, Rzeszow University of Technology, Powstancow Warszawy 12, 35-959 Rzeszow, Poland; 3 Electrical Engineering Department, King Khalid University, Abha 61411, Saudi Arabia; amayet@kku.edu.sa (A.M.M.); hloukil@kku.edu.sa (H.L.); rmanoharan@kku.edu.sa (M.R.R.); mabdulmuqeet@kku.edu.sa (M.A.M.); 4 Department of Energy, Universidad de la Costa, Barranquilla 080001, Colombia; 5 Institute of Optics and Quantum Electronics, Friedrich Schiller University Jena, Max-Wien-Platz 1, 07743 Jena, Germany

**Keywords:** 8-electrode sensor, measuring, temperature, pressure, artificial intelligence, air-water homogenous regime

## Abstract

Two-phase fluids are widely utilized in some industries, such as petrochemical, oil, water, and so on. Each phase, liquid and gas, needs to be measured. The measuring of the void fraction is vital in many industries because there are many two-phase fluids with a wide variety of liquids. A number of methods exist for measuring the void fraction, and the most popular is capacitance-based sensors. Aside from being easy to use, the capacitance-based sensor does not need any separation or interruption to measure the void fraction. In addition, in the contemporary era, thanks to Artificial Neural Networks (ANN), measurement methods have become much more accurate. The same can be said for capacitance-based sensors. In this paper, a new metering system utilizing an 8-electrode sensor and a Multilayer Perceptron network (MLP) is presented to predict an air and water volume fractions in a homogeneous fluid. Some characteristics, such as temperature, pressure, etc., can have an impact on the results obtained from the aforementioned sensor. Thus, considering temperature changes, the proposed network predicts the void fraction independent of pressure variations. All simulations were performed using the COMSOL Multiphysics software for temperature changes from 275 to 370 degrees Kelvin. In addition, a range of 1 to 500 Bars, was considered for the pressure. The proposed network has inputs obtained from the mentioned software, along with the temperature. The only output belongs to the predicted void fraction, which has a low MAE equal to 0.38. Thus, based on the obtained result, it can be said that the proposed network precisely measures the amount of the void fraction.

## 1. Introduction

In various industrial sectors, such as the chemical, petrochemical, oil, and gas industries, two-phase flows involving combinations such as air-oil, water-oil, and air-water are commonly encountered [1]. 

Accurately measuring the void fraction in these diverse fluid systems is a critical challenge [2]. Flow measurement holds significant importance in industries for financial metering, process control, storage management, and other applications. Due to the complexities associated with multi-phase fluids, accurately gauging such flows are challenging [3]. Conventional methods that separately gauge the amount of every fluid’s phase are proven to be time-consuming and costly [4]. Consequently, it is necessary to develop a flow gauge that can identify the flow type and measure its volume fraction inside the pipe without causing disruption to ongoing processes [5,6]. 

To assess the volume of the air phase in any flow comprising gas (or air) and liquid, a common approach is partitioning the phase containing air by the whole volume of the flow. This technique enables the quantification of the flow’s volume phase. Multiple methods exist for determining the void fraction, including radiation attenuation, ultrasonic wave-based approaches, capacitance-based impedance measurements, wire mesh sensors, and assessing volume through valve manipulation [5,6,7,8,9,10,11]. 

Among these techniques, capacitance-based sensors offer a promising solution for void fraction measurement, eliminating the requirement to detach the phases or disturb the ongoing operation. The arrangement of electrodes is vital for this kind of sensor, and the selection of an appropriate electrode configuration greatly influences the accuracy of the measurements. The choice of electrode arrangements, such as concave, ring, or helix, depends on the fluid properties during transportation through the tube. Previous investigations have primarily focused on two-phase flows within pipes: stratified, annular, and homogeneous flows [12,13,14,15]. 

In a study conducted by Li and colleagues [16], they explored the error-measuring matter in capacitance-based sensors and identified that the incorporation of homogeneous sensibility can effectively decrease such errors. Subsequent endeavors were undertaken to augment and establish a framework that ensures uniform sensitivity, wherein it was ascertained that the helical electrode is the most efficacious means of attaining said objective [17,18,19]. An investigation was performed by Tollefsen and his co-workers [19] on a two-phase, water and oil, system and found that the implementation of capacitive sensors using direct plate surfaces is restricted by the regime and redistribution. In order to obtain precise outcomes, it is of utmost importance to amalgamate the various flow components. In instances where the dimensions of the bubbles are inferior to those of the matter, the resultant mixture exhibits a state of near-homogeneity, whereby the individual constituents intermingle to a significant degree. In a separate examination [20], some shapes, such as helix, concave, and double ring, were scrutinized in an air and water scenario. Among the aforementioned options, the second showcased unparalleled sensitivity. In previous investigations, the utilization of capacitance sensors to quantify fluids consisting of dual-phase compositions has been subjected to scrutiny, revealing a range of sensitivity levels exhibited by said sensors. For instance, researchers noted [21] that while the concave structure demonstrated the utmost sensitivity in a water-air flow with two phases, among the examined configurations, it was observed that the double-ring arrangement was the least sensitive. As deduced from the findings of a previous study [22], where a comprehensive evaluation of various electrode types, including concave, parallel plate, ring, and helical, within an air and solid flow was conducted, it was ascertained that the concave sensor exhibited the utmost degree of sensitivity. Sami and Aboulwafa [23] delved into the treatment of a non-conductive liquid and air dual-phase flow. By undertaking a series of experiments involving six discrete capacitors, researchers were able to discern that the helical sensor, when employed for the detection of air-oil fluid, showcased unparalleled sensitivity. Furthermore, their investigation revealed that the concave sensor excelled in accurately identifying the annular regime, rendering it the most efficacious in this particular context. In his study, Ahmed [24] utilized a capacitive sensor to discern the volume fraction and discern the fluid type within a horizontally positioned pipeline conveying a dual-phase air-oil mixture. The sensitivity of the capacitive sensor was thoroughly assessed by employing concave and ring electrodes, which revealed that the ring electrode exhibited superior sensitivity compared to the concave electrode. Roshani et al. [25] conducted a comparative analysis between two widely used sensors. The sensor technologies under consideration encompass capacitance-based sensors and gamma-ray attenuation sensors, which employ the attenuation of gamma radiation for measurement purposes. The investigation was carried out in a scenario involving an annular flow of air and oil. The outcomes of the investigation unveiled that, within the void fraction range of 0.8 to 1, the concave sensor demonstrated superior performance when compared to the gamma-ray attenuation sensor in accurately ascertaining void fractions. In a comprehensive study conducted by Chen et al. [26], an examination of a two-phase flow was undertaken, employing a diverse series of sensors, including concave, double-ring, and array sensors. The primary aim of their endeavor was to quantify the void fraction while simultaneously unraveling aspects pertaining to flow dynamics. In their study, Jaworek et al. [27] employed a sensor resembling the concave sensor configuration to ascertain the void fraction within confined channels featuring diameters that were less than 10 mm. To accomplish this, in order to evaluate the void fraction in the two-phase flow, the researchers established a connection between the aforementioned sensor and an intricate arrangement of components as a resonant circuit equipped with a parallel inductance. The analysis of the high-frequency oscillator’s frequency deviation served as the basis for assessing the void fraction. In their comprehensive investigation, Bai et al. [28] quantified the void fraction in stratified air-liquid flow. To accomplish this, they employed a capacitance probe that boasted multiple wires, thus expanding upon the capabilities of the single-wire capacitance probe. With the aid of the multi-wire probe, the researchers were able to assess both the average and local volume fractions by meticulously observing the variation in water layer height at distinct circumferential positions within the tube network. 

The proliferation of ANN as a tool has experienced a substantial surge in popularity spanning diverse disciplines, including but not constrained solely to the domains of electrical engineering and control engineering [29,30,31,32]. 

Material properties such as temperature, pressure, and many others highly affect the results obtained. That is why the aforementioned characteristics must be considered during the measuring process. In light of these considerations, this paper proposes an innovative methodology to deviate from conventional practices and utilizes novel avenues of inquiry to harness the transformative potential of artificial intelligence (AI) to meticulously ascertain the volume fraction within a multi-phase flow with unparalleled precision. There are many applications related to AI, and a number of those previously studied were conducted through machine learning. In this regard, the modeling of electrohydrodynamic pumps, bidirectional electrohydrodynamic pumps, and fabric-type actuators can be mentioned [33,34,35]. 

The employed methodology revolves around training the AI algorithm using an intricately crafted data set generated through the utilization of the COMSOL Multiphysics software. The simulations conducted entailed the modeling of a capacitance-based sensor, which was an 8-electrode sensor. The simulations were performed on a water and air homogeneous flow. 

One of the pivotal characteristics of the fluid investigated was the static dielectric constant, commonly referred to as the relative permittivity (ε_r_), which was explored under varying fluid characteristics such as pressure and temperature. This parameter holds significant importance, exerting influence on the solvent behavior of water across diverse environments and encompassing an expansive range of applications spanning both biological and industrial domains [36]. Over the years, scientists have devised a number of mathematical formulations aimed at accurately forecasting the water’s ε_r_, notable contributions have been made, utilizing the formulation proposed by Quist and Marshall, emerging as a pioneering advancement within this field [37]. Continued efforts have been dedicated to refining experimental outcomes, expanding the exploration of temperature and pressure ranges, and proposing exploratory formulations aimed at providing alternatives to enhance the accuracy of predictions. Fernandez and colleagues [38] compiled an exhaustive database encompassing the entirety of the accessible data on water’s ε_r_. With meticulous attention, they embarked upon a comprehensive evaluation of the diverse methodologies employed to deduce the ε_r_, they diligently scrutinized the available data and handpicked the most precise subset to be incorporated into the data correlation process. Leveraging a specific portion of this selected subset, they introduced an innovative formulation that integrates a statistical regression function, enabling a precise approximation of the ε_r_ within the entire range of temperature and pressure encompassed by the experimental observations. Their study provided a comprehensive compilation of water’s ε_r_ covering a temperature spectrum ranging from 270 to 375 Kelvin and a pressure range spanning from 1 to 500 Bars. 

The current investigation incorporates the data extracted from the COMSOL Multiphysics software, derived from an 8-electrode sensor with different states. These collected data, along with the temperature, served as inputs to train the proposed neural network known as a Multilayer Perceptron network (MLP), implemented within the MATLAB environment. Recent studies have also delved into the optimization of such sensors through the application and exploitation of ANN [39,40,41]. 

The primary aim of this endeavor is to quantitatively determine the void fraction considering the temperature changes within a homogeneous two-phase system consisting of air and water, ensuring its independence from variations in pressure conditions. In essence, the study introduces a metering system capable of accurately predicting the void fraction, regardless of the pressure conditions. To attain this crucial objective, an essential step involved the collection of data. Consequently, the COMSOL Multiphysics software was employed to conduct simulations utilizing an 8-electrode sensor. The resulting data were then utilized as inputs for the proposed MLP ANN. This MLP model, which leverages a novel and precise predicting system, exhibited the capability to predict the void fraction with a remarkable degree of accuracy while minimizing error.

The novelty of the presented metering system is that it measures the volume fraction using an 8-electrode sensor and an ANN, considering temperature changes and independent of pressure variations for a two-phase homogeneous fluid.

## 2. Validating and Simulations

**Figure 1:**
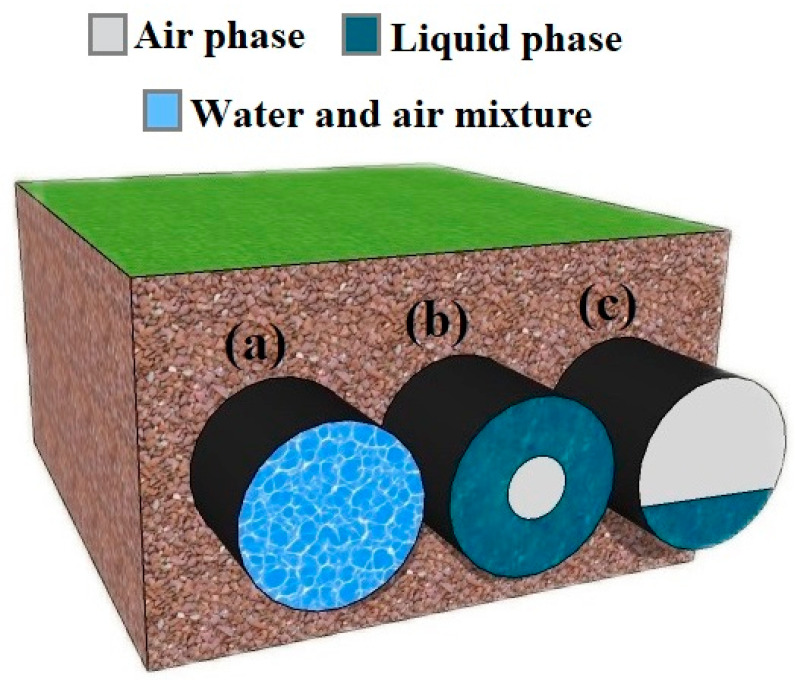
Main regimes in industries. (**a**) Homogeneous, (**b**) annular, and (**c**) stratified.

In this section, a homogeneous regime is implemented in the COMSOL Multiphysics software to be investigated by an 8-electrode sensor. This software is useful in designing and implementing capacitance-based sensors. Three main regimes exist in some industries, such as oil, petrochemicals, water, and so on. These fluids as homogeneous, annular, and stratified fluids are shown in Figure 1a–c, respectively. In the authors’ previous works [42], numerous experiments were conducted to benchmark the aforementioned software. The outcomes derived from testing both the physically manufactured sensor and its simulated counterpart exhibited strikingly similar trends, with only minimal variance in the error rates. When two phases of fluid, air and water, are mixed thoroughly, a homogeneous regime is created. To implement this kind of fluid in the software, by averaging the relative permittivity of two phases in every volume fraction, the mentioned regime can be created.

In the following, the designed 8-electrode sensor is investigated in detail. In this study, the principal objective lies in the precise measurement of the void fraction within a homogeneous system of air and water, eliminating any dependence on pressure fluctuations, considering the temperature. The fact that there is a definite link between the behavior of a liquid and its relative permittivity is obvious. Thus, it is essential for the amount of water’s ε_r_ to be in different ranges of temperature and pressure. In pursuit of attaining the desired quantities, the presented numbers in [38] are used for the simulations in this paper. The utilized ε_r_s are presented in Table 1 for a range of temperatures from 275 to 370 degrees Kelvin. In addition, a range of 1 to 500 Bars has been considered for the pressure. As can be seen in Table 1, there are 9 and 20 different numbers for the pressure and temperature, respectively. For any situation, two various characteristics are considered, and then a specific amount of ε_r_ exists. For instance, when the temperature and pressure are equal to 300 degrees centigrade and 100 Bars, respectively, the ε_r_ is equal to 78.11. To implement different volume fractions in the software, after knowing the ε_r_ of water, by averaging both phases’ relative permittivity, the correct amount of ε_r_ is considered. It is to be noted that there is a direct relationship between the pressure and the water’s ε_r_. Conversely, there is not any kind of direct relationship between the temperature changes and the ε_r_ of water; the higher the temperature, the less relative permittivity.

**Figure 2:**
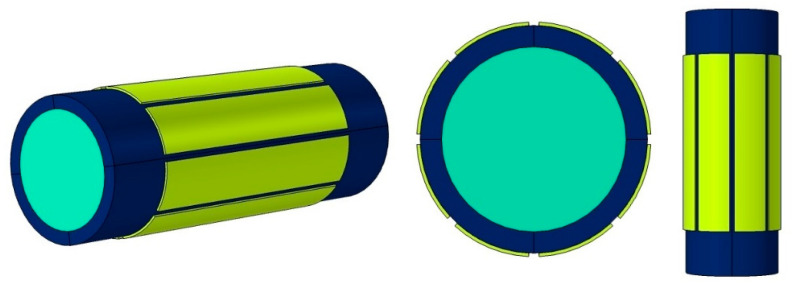
Different views of the 8-electrode sensor.

**Figure 3:**
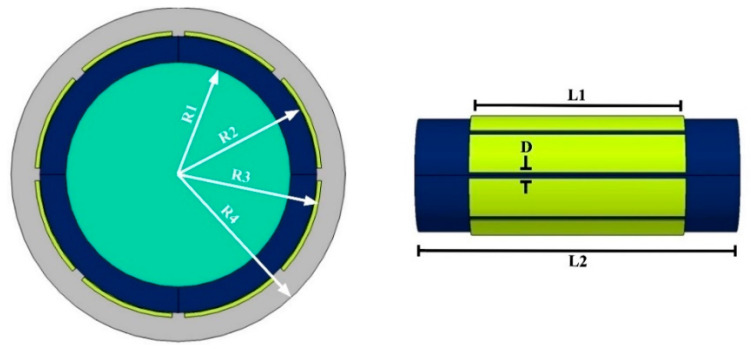
Dimensions of the 8-electrode sensor.

Some kinds of sensors exist and are popular such as concave, ring, and helix. The designed sensor contains eight electrodes and is close to the common concave sensor. The difference is that the 8-electrode sensor gives this opportunity to obtain more results, which can help provide more inputs to train the network. Various views of the designed 8-electrode sensor are presented in Figure 2. The liquid phase, pipe, and electrodes are recognizable with light blue, dark blue, and yellow colors, respectively. In Figure 3, the dimensions of the different parts of the 8-electrode sensor are named. The lengths of the electrodes and pipe are equal to L1 = 12 cm and L2 = 18 cm, respectively. Moreover, the distance between the electrodes is equal to D = 0.3 cm. In addition, about the other parts, the inner radius, the outer radius, the outer radius of the electrodes, and the radius of the isolated area in the software are equal to R1 = 2.6 cm, R2 = 3.2 cm, R3 = 3.3 cm, and R4 = 5 cm, respectively. For electrodes, a copper plate with a high conductivity and, of course, a thickness equal to 0.1 cm was utilized. 

**Figure 4:**
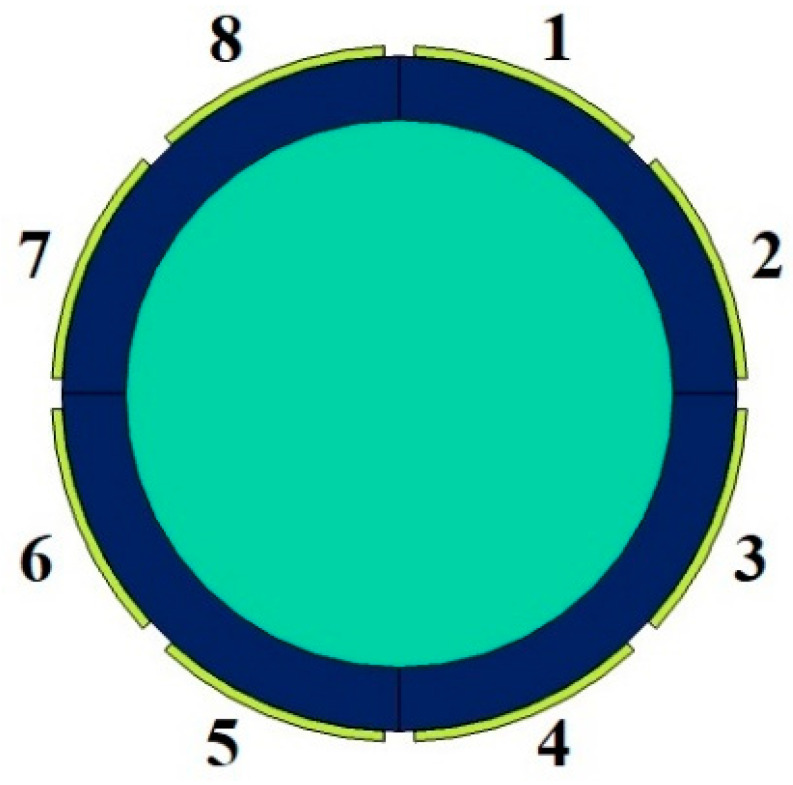
The appellation of different electrodes of the 8-electrode sensor.

**Figure 5:**
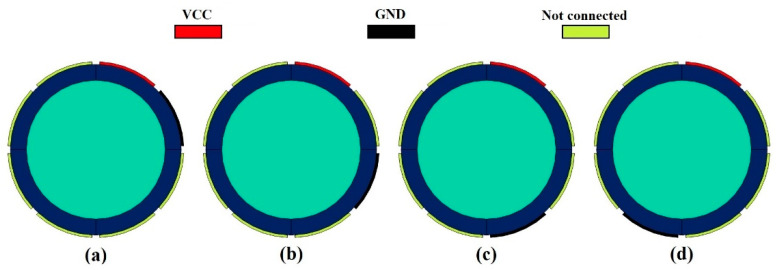
Different states of measuring, (**a**) 1–2, (**b**) 1–3, (**c**) 1–4, and (**d**) 1–5.

**Figure 6:**
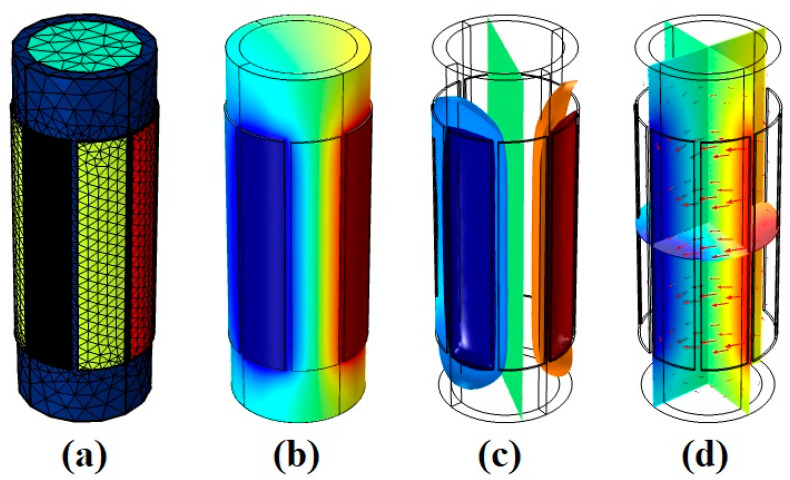
Some models of the 8-electrode sensor in the 1–3 state of measurement, (**a**) mesh, (**b**) volume, (**c**) isosurface, and (**d**) multislice, along with arrow surface.

As mentioned before, the 8-electrode sensor can produce more results, which are helpful for training networks. Thus, each of the eight electrodes is named and determined in Figure 4. To produce data, every pair of electrodes was considered and simulated in the COMSOL Multiphysics 180 times (there were 9 and 20 different settings for the pressure and temperature, respectively). As Figure 5 shows, there are four different states for measuring the capacity between electrodes 1, 2, 3, 4, and 5, which are named 1–2, 1–3, 1–4, and 1–5 states, respectively. Some models are given in Figure 6, which belong to mesh, volume, isosurface, and multislice, along with arrow surface, respectively. After simulating all the aforementioned states for all ranges of temperature and pressure, 3780 different data points were extracted. For the proposed network, 1134 data points were considered as test data, 50 of which are given in Table 2 as samples.

## 3. Artificial Neural Network

**Figure 7:**
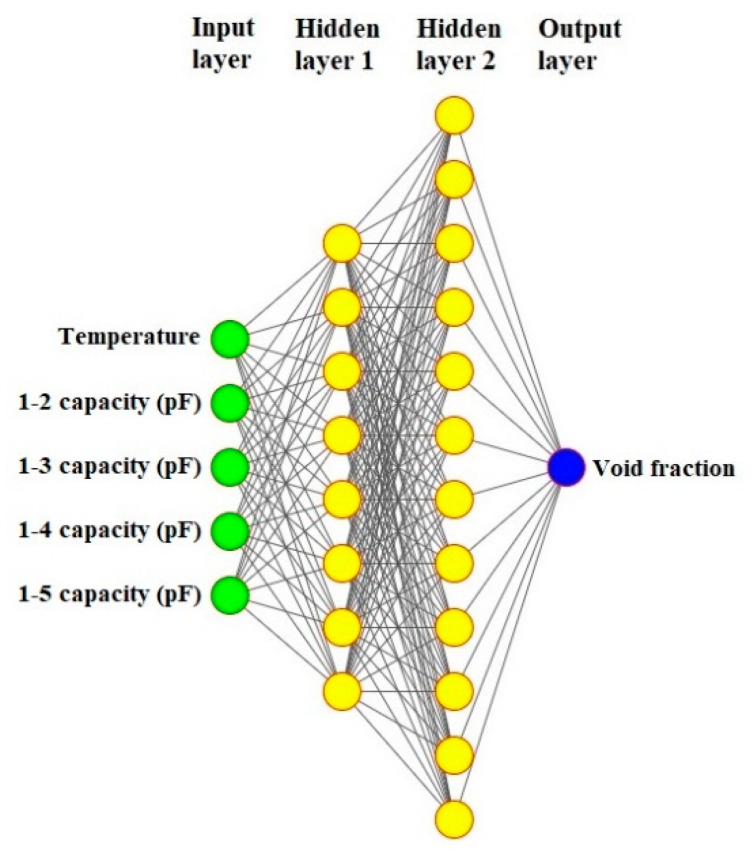
The architecture of the presented network.

**Figure 8:**
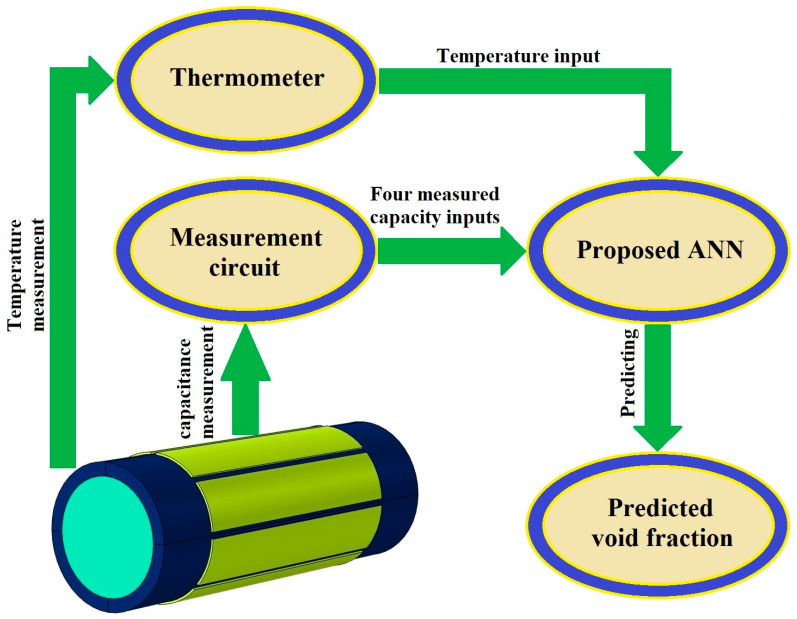
Visual representation of the proposed metering system utilizing an 8-electrode sensor and an ANN.

The concept of ANN has held a significant position in the field of artificial intelligence since the 1980s. At its core, ANN is designed to abstract and mimic the intricate information processing of the human brain’s neural network. Through this abstraction, a simplified model is established that is capable of being adapted into diverse network configurations, achieved by employing varying connections [43]. The primary aim is to faithfully replicate the brain’s neural network processing and effectively store memory information using sophisticated information processing techniques. Within both engineering and academic circles, these networks are commonly referred to simply as neural networks. Functionally, a neural network operates as a powerful computational model characterized by a multitude of interconnected nodes, or neurons [44]. Each individual node is associated with a specific output function, known as the activation function. Meanwhile, the connections linking these nodes are defined by weight factors, denoting the strength of signal transmission along these interconnections. It is through these weights that the artificial neural network effectively manages its memory [45]. Crucially, the network’s ultimate output is dictated by its architectural design, the precise values assigned to the interconnected weights, and the activation functions employed. Consequently, the neural network frequently approximates complex algorithms or natural functions and can even express intricate logical strategies [46]. The realm of artificial intelligence (AI) exhibits a vast array of applications across diverse sectors [47,48,49]. This tool is an exquisitely refined mathematical method that utilizes computing elements known as neurons, adroitly arranged in a manner that encompasses singular or manifold layers of computational prowess [50]. The ANN framework exhibits different kinds of useful networks, each harboring a distinct feature. Among these, the multilayer perceptron (MLP) emerges as a preeminent exemplar, having garnered widespread recognition for its unswerving precision and extraordinary capacity to approximate data points with utmost fidelity [51]. The MLP model encapsulates two indispensable datum subsets, called the train set and the test set. The first one constitutes a limited pool of meticulously curated datum points, adroitly employed to train the model’s cognitive faculties, whereas the latter embodies unfamiliar data that serves as an evaluative metric to gauge the network’s efficacy and accuracy [52]. To achieve the optimal ANN configuration that has a low Mean Absolute Error (MAE), an exhaustive suite of networks varying across a panorama of architectural attributes, including epoch quantities, hidden layers, and activation functions, were painstakingly evaluated. Ultimately, the best configuration, as a novel and optimal metering methodology, was presented. In the present study, the MLP network, conceived as a meticulous model, has a 5-input configuration, wherein the capacities obtained from the 8-electrode sensor and the temperature act as inputs. In Table 2, there are 50 different rows as test data, and each row belongs to each temperature, pressure, void fraction, relative permittivity, and the obtained capacities of all four different states. Through the employment of COMSOL Multiphysics software, an exhaustive repertoire of 180 simulations was meticulously orchestrated, encompassing diverse temperature and pressure ranges, while effectuating incremental modifications to void fractions spanning the gamut from 0 to 100 percent, in which 5 percent was incremented. From this assemblage of simulations, 2646 cases (constituting 70% of the aggregate) were randomly earmarked as the train data, while 1134 cases (constituting 30% of the aggregate) were sequestered as test data. Following extensive experimentation involving the testing of diverse network configurations that encompassed different numbers of neuron quantities and stratified layers, the configuration that proved to have the lowest MAE emerged as the definitive network, with its detailed specifications outlined in Table 3. The architecture of the proposed network and visual representation of the proposed metering system are portrayed in Figure 7 and Figure 8, respectively. In the quest for the most suitable network architecture, extensive exploration has been undertaken to identify the network exhibiting the lowest mean absolute error. Diverse network configurations have been meticulously examined, encompassing variations in crucial parameters including the number of neurons, epochs, hidden layers, and even the activation functions employed. Through a rigorous and iterative process, various components of the network were meticulously scrutinized, modified, and adjusted until arriving at the ultimate selection of the proposed network. The culmination of this meticulous investigation led to the identification of the network that displayed superior performance. As it is clear from Figure 8, after measuring the various capacities of different states and the temperature, the results are given as inputs to the presented network. This way, the model predicts the amount of void fraction.

## 4. Results and Discussion

**Figure 9:**
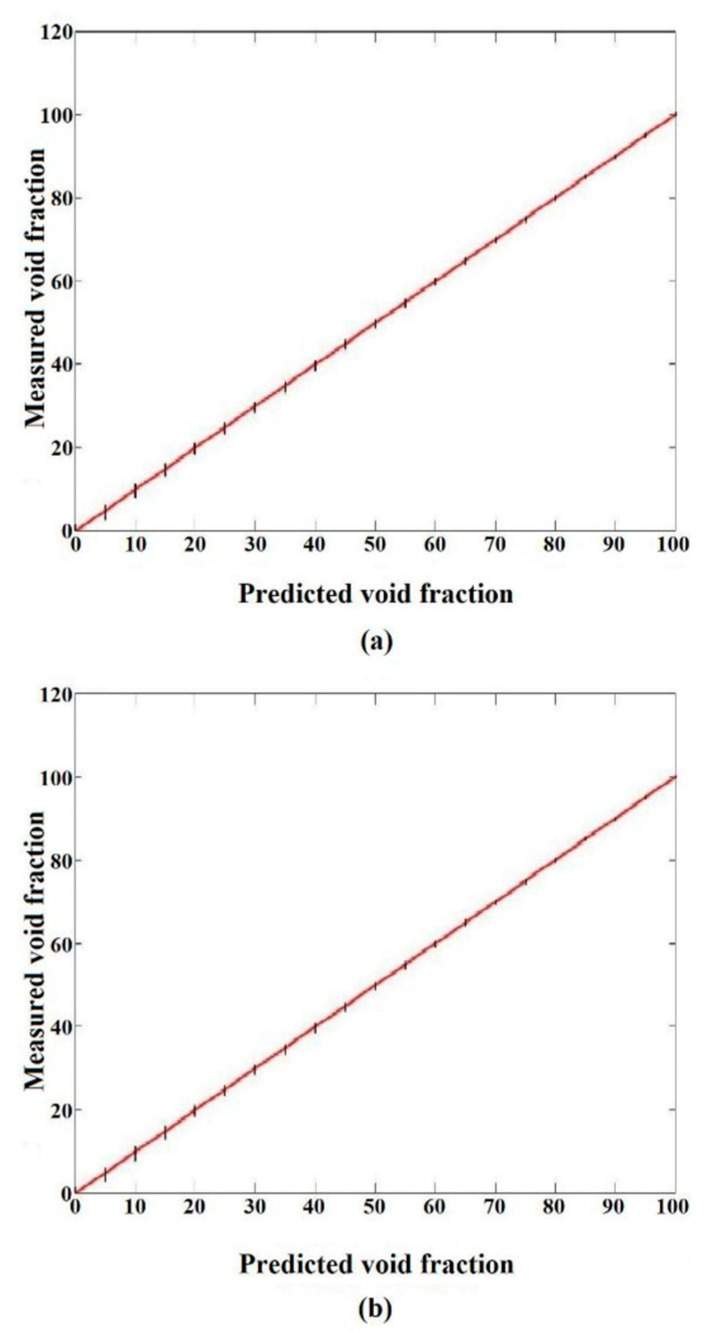
Obtained results from the 8-electrode sensor. (**a**) Training data and (**b**) test data.

Material properties such as temperature, pressure, and many others highly affect the accuracy of the obtained results. This is why the aforementioned characteristics have to be considered during the measurement process. In light of these considerations, simulations were performed by the COMSOL Multiphysics software in different ranges of temperature and pressure. These characteristics affect the water’s ε_r_ and this issue highly impacts the accuracy of capacitance-based sensors. So, it is obvious that there is a clearly recognizable correlation between these considerations and the attainment of precise measurements in practical applications. To produce more data related to the liquid passing through the tube, an 8-electrode sensor was utilized. The resulting data from this sensor (1–2, 1–3, 1–4, and 1–5 states) were then fed into an ANN for predicting the void fraction with maximum accuracy. It is important to note that, despite each state possessing its own specific capacity, the objective of this investigation is to predict the void fraction independent of fluctuations in the pressure variation, considering the temperature changes. Consequently, the design of the measuring electrodes was meticulously crafted with the aim of obtaining as much detail as possible related to the liquid phase for training the presented ANN as well as possible. Volume fraction measurement using the introduced network is illustrated in Figure 8. As previously mentioned, a total of 3780 data points were accumulated through simulations employing COMSOL Multiphysics. Among these outcomes, 70% of the total (2646 cases) were allocated for training the proposed network, while the remaining 1134 data points (30% of the total) were reserved for testing the performance of the network. This division of data was performed randomly, ensuring a fair distribution across the training and test sets. The optimal structure of the network was determined over numerous assays, wherein various networks with diverse characteristics were evaluated. Figure 9 shows the regression diagrams for both groups of data, illustrating the outcomes of the proposed model. Compared to previous related studies, recently in [41], a homogeneous regime was investigated, which reported the MAE equal to 4.723 and 4.868 for the train and test data, respectively. Due to the use of an optimized sensor, a more appropriate model, and more inputs compared to the mentioned study, the current network demonstrated much better performance. Although there was a vast collection of 3780 distinct data points, a considerable portion of them were in remarkable proximity, which led to a low amount of error, the presented network was accurate. For both data sets, the train and test, the MAE values are determined as 0.36 and 0.38, respectively. The objective of this research was to gauge the void fraction considering temperature and variation independent of pressure changes within a homogeneous fluid consisting of air and water. The presented measuring system demonstrated a low MAE, indicating a high level of accuracy.

As previously indicated, the ANN is exposed to two distinct types of data: the train data and the test data. The training data serves as the foundation for instructing and constructing the network model, encompassing information that the network processes during its training phase. Subsequently, the trained model undergoes rigorous testing. During this evaluation process, the predicted values of both the training and test data are compared against the corresponding real values from their respective datasets. Notably, the comparison reveals a noteworthy absence of over-fitting and under-fitting issues associated with the network under consideration.

## 5. Conclusions

In this study, a new metering system was presented to measure the amount of void fraction. Measuring fractions is vital in a number of industries. So, the presented system was utilized for an air and water homogeneous regime. An MLP ANN was created to achieve the aforementioned goal. This network had five inputs; four different inputs were obtained from simulating the 8-electrode sensor in COMSOL Multiphysics and another input was the temperature ranging from 275 to 370 degrees Kelvin. Based on every different amount for both temperature and pressure, there were various amounts of the ε_r_ of the liquid phase of the two-phase fluid, which was water. The simulations iterated a total of 180 times, resulting in the collection of an extensive dataset comprising 3780 distinct data points, 70 percent of which were utilized as training data and the remaining used as test data. The proposed neural network exhibited the capability to quantitatively assess the void fraction regardless of variations in pressure magnitude (from 1 to 500 Bar), thereby exhibiting its independence from pressure effects with a very low MAE equal to 0.38.

## Figures and Tables

**Table 1:** Impact of temperature and pressure changes on the ε_r_ of water [38].

	**Pressure (Bar)**									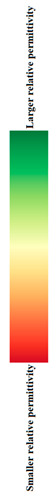
**Temperature (Kelvin)**	**1**	**10**	**20**	**50**	**100**	**200**	**300**	**400**	**500**	
275	87.16	87.2	87.24	87.36	87.57	87.97	88.38	88.77	89.16	
280	85.19	85.23	85.27	85.39	85.59	85.98	86.37	86.76	87.14	
285	83.27	83.3	83.34	83.46	83.65	84.04	84.42	84.8	85.17	
290	81.39	81.42	81.46	81.57	81.76	82.14	82.51	82.88	83.24	
295	79.55	79.58	79.62	79.73	79.92	80.29	80.65	81.01	81.37	
300	77.75	77.78	77.82	77.93	78.11	78.48	78.83	79.19	79.54	
305	75.99	76.02	76.06	76.17	76.35	76.71	77.06	77.41	77.75	
310	74.27	74.3	74.33	74.44	74.62	74.98	75.32	75.67	76.01	
315	72.58	72.61	72.65	72.76	72.93	73.28	73.63	73.97	74.3	
320	70.93	70.97	71	71.11	71.28	71.63	71.97	72.31	72.64	
325	69.32	69.36	69.39	69.5	69.67	70.02	70.35	70.69	71.01	
330	67.75	67.78	67.82	67.92	68.09	68.44	68.77	69.1	69.43	
335	66.21	66.24	66.27	66.38	66.55	66.89	67.23	67.55	67.88	
340	64.7	64.73	64.77	64.87	65.04	65.38	65.72	66.04	66.36	
345	63.23	63.26	63.3	63.4	63.57	63.91	64.24	64.56	64.88	
350	61.79	61.82	61.85	61.96	62.13	62.47	62.8	63.12	63.44	
355	60.38	60.41	60.45	60.55	60.72	61.06	61.39	61.71	62.03	
360	59	59.03	59.07	59.17	59.34	59.68	60.01	60.33	60.65	
365	57.66	57.69	57.72	57.83	58	58.34	58.67	58.99	59.3	
370	56.34	56.37	56.41	56.51	56.68	57.02	57.35	57.67	57.99	

**Table 2:** Some of the test data which were obtained from all available states of the 8-electrode sensor.

**T(K)**	**Pressure (Bar)**	**Void Fraction**	**ε_r_**	**1–2 (pF)**	**1–3 (pF)**	**1–4 (pF)**	**1–5 (pF)**	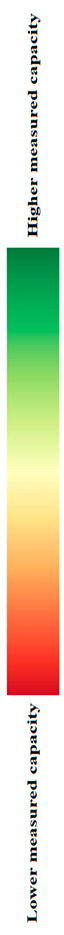
305	500	0	77.75	10.185	8.1185	7.9675	7.9329	
365	50	70	18.049	9.1278	6.5335	6.1916	6.1068	
335	500	0	67.88	10.128	8.0269	7.8622	7.824	
295	100	30	56.244	10.036	7.8831	7.6978	7.6539	
315	500	30	52.31	9.9968	7.8222	7.6283	7.5821	
345	50	40	38.44	9.8042	7.526	7.2927	7.2359	
365	50	100	1	5.7216	2.4351	1.9867	1.8844	
305	20	60	31.024	9.643	7.2829	7.0197	6.955	
275	500	0	89.16	10.238	8.2014	8.0629	8.0319	
315	300	20	59.104	10.061	7.923	7.7434	7.701	
365	1	0	57.66	10.049	7.9033	7.7209	7.6777	
285	1	50	42.135	9.8662	7.6207	7.3997	7.3461	
325	500	70	22.003	9.3343	6.829	6.5157	6.438	
365	300	70	18.301	9.1429	6.5549	6.215	6.1306	
305	1	60	30.996	9.6423	7.2818	7.0185	6.9537	
365	20	60	23.688	9.406	6.933	6.6305	6.5556	
335	300	60	27.492	9.5416	7.1321	6.8515	6.7822	
325	20	0	69.39	10.137	8.0424	7.88	7.8424	
365	400	60	24.196	9.426	6.9622	6.6629	6.5888	
335	100	100	1	5.7216	2.4351	1.9867	1.8844	
325	10	0	69.36	10.137	8.0421	7.8797	7.842	
325	1	50	35.16	9.74	7.4286	7.1831	7.123	
365	200	40	35.404	9.7451	7.4364	7.1918	7.132	
345	100	0	63.57	10.097	7.979	7.8074	7.7672	
295	400	20	65.008	10.108	7.9956	7.8264	7.7869	
325	200	60	28.608	9.5758	7.1829	6.908	6.8402	
275	20	80	18.248	9.1398	6.5505	6.2101	6.1256	
365	500	40	35.98	9.7569	7.4543	7.212	7.1527	
365	50	0	57.83	10.05	7.9057	7.7236	7.6805	
285	200	0	84.04	10.216	8.1667	8.023	7.9904	
305	10	70	23.506	9.3986	6.9223	6.6187	6.5435	
345	200	70	19.873	9.2308	6.68	6.3519	6.2705	
325	1	0	69.32	10.137	8.0417	7.8792	7.8416	
295	200	0	80.29	10.198	8.1388	7.9908	7.9571	
355	50	10	54.595	10.02	7.8585	7.6697	7.6249	
335	20	70	20.581	9.267	6.7319	6.4089	6.3288	
345	300	0	64.24	10.102	7.9868	7.8164	7.7765	
295	20	70	24.586	9.441	6.9841	6.6871	6.6136	
295	300	20	64.72	10.106	7.9924	7.8227	7.783	
305	300	10	69.454	10.138	8.043	7.8808	7.8432	
335	50	80	14.076	8.8407	6.1331	5.7573	5.6642	
345	400	40	39.136	9.8166	7.545	7.3141	7.2579	
325	10	40	42.016	9.8644	7.6179	7.3965	7.3428	
345	1	90	7.223	7.9558	4.9702	4.5265	4.4184	
285	1	90	9.227	8.2943	5.403	4.9793	4.8753	
345	50	80	13.48	8.7878	6.0607	5.6793	5.5849	
345	500	60	26.552	9.511	7.0869	6.8012	6.7306	
305	1	20	60.992	10.077	7.9476	7.7715	7.73	
355	400	30	43.497	9.8868	7.6522	7.4353	7.3829	
355	200	10	55.054	10.025	7.8655	7.6777	7.6331	

**Table 3:** Configuration of the presented MLP ANN model.

Neural Network	MLP
Number of neurons in the input layer	5
Number of neurons in hidden layer 1	8
Number of neurons in hidden layer 2	12
Number of neurons in the output layer	1
Number of epochs	600
Activation function of neurons in hidden layer 1	Tansig
Activation function of neurons in hidden layer 2	Tansig
Activation function of neurons in the input and output layers	Purelin
Method of training	Levenberg-Marquardt [53,54]

## Data Availability

The data presented in this study are available on request from the corresponding author.
